# Electrolyte-Controlled Self-Assembly of Water-Soluble Perylene Bisimides: A Kinetic and Stimulated Raman Study

**DOI:** 10.1021/acs.jpclett.6c02220

**Published:** 2026-08-28

**Authors:** Miles Martinati, Giovanni Batignani, Gaia De Maria, Giancarlo Fabrizi, Yuri Gazzilli, Valeria Pennacchietti, Fang Yang, Alberto Boffi, Tullio Scopigno, Roberta Piacentini

**Affiliations:** † Department of Physics, 9311 Sapienza University of Rome, Piazzale Aldo Moro 5, 00185 Rome, Italy; ‡ Department of Chemistry and Pharmaceutical Technologies, 9311 Sapienza University of Rome, Piazzale Aldo Moro 5, 00185 Rome, Italy; § Department of Biochemical Sciences “Alessandro Rossi Fanelli”, 9311 Sapienza University of Rome, Piazzale Aldo Moro 5, 00185 Rome, Italy; ∥ Ningbo Key Laboratory of Biomedical Imaging Probe Materials and Technology, Laboratory of Advanced Theranostic Materials and Technology, Chinese Academy of Sciences (CAS) Key Laboratory of Magnetic Materials and Devices, Ningbo Institute of Materials Technology and Engineering, Chinese Academy of Sciences, Ningbo 315201, China; ⊥ Zhejiang International Cooperation Base of Biomedical Materials Technology and Application, Zhejiang Engineering Research Center for Biomedical Materials, Ningbo Cixi Institute of Biomedical Engineering, Cixi 315300, China; # D-Tails s.r.l. S.B., Via Agrigento, 4A/4B, 00161 Rome, Italy

## Abstract

Perylene bisimides (PBIs) are prime building blocks for functional supramolecular architectures, yet mapping their salt-induced self-assembly kinetics under physiological conditions remains challenging, due to rapid aggregation and overwhelming fluorescence. Here, we combine fluorescence stopped-flow kinetics and stimulated Raman scattering (SRS) spectroscopy to decipher the electrolyte-controlled polymerization of a spermine-functionalized PBI. We found that self-assembly obeys an anticooperative K2–K pathway, where nucleation yields rapid and salt-independent dimers. In contrast, polymer growth is strongly salt-dependent, with polymer dissociation rates decreasing as NaCl concentration increases. Crucially, chloride ions selectively stabilize the growing assemblies by drastically suppressing the monomer release, establishing a Michaelis–Menten-type saturation profile. To link these kinetic trajectories with molecular-scale structural rearrangements, SRS is used to effectively suppress the fluorescence background, revealing that the relative intensity of low-frequency Raman modes, associated with high-mass displacement and structurally delocalized in nature, are drastically reduced upon aggregation. This vibrational suppression underscores the role of packing constraints and excitonic pressure within the cofacial H-aggregates. By directly bridging macroscale kinetic pathways with microscopic vibrational dynamics, this work offers a generalizable framework to rationally design and tune water-soluble supramolecular materials for biomedical and optoelectronic applications.

## Graphical abstract

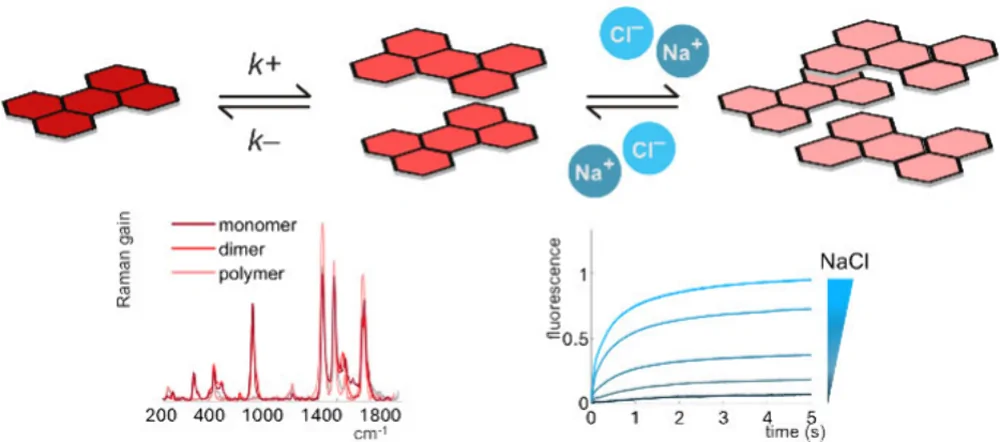

In fluorescence-based biochemical and cell biology applications, fluorophores must combine high aqueous fluorescence quantum yield, suitable absorption and emission bands, chemical and photochemical stability under physiological conditions, low toxicity, and, ideally, monodispersity in aqueous solutions.^1–3^ Perylene bisimides, also commonly referred to as perylene diimides (PBIs/PDIs), are derivatives of the perylene-3,4,9,10-tetracarboxylic diimide scaffold. In this context, PBIs have emerged as highly attractive fluorescent labels, because they satisfy many of these requirements.^4–6^ However, their rigid planar cores and extended π-conjugation strongly promote intermolecular π–π stacking, leading to aggregation in aqueous media, particularly in the presence of salts.^7,8^ Electron microscopy and AFM studies have shown that PBIs can form columnar one-dimensional nanostructures, which may further evolve into higher-order assemblies, depending on the imide substituents.^9–14^ This aggregation tendency limits the use of PBIs in live-cell imaging and biochemical assays requiring physiological buffers, while also making them valuable model systems for studying supramolecular self-assembly.^8,14^ Their aggregation behavior has been extensively investigated by UV–visible absorption and fluorescence spectroscopy, exciton theory, and quantum mechanics/molecular mechanics/molecular dynamics simulations.^15–18^ These studies have clarified key features such as exciton splitting and “non-Franck-Condon” vibronic progressions in H- and J-type aggregates.^17,19^

The self-assembly of PBIs is primarily governed by the chemical nature of their peripheral substituents, which control solubility and modulate the balance between π–π stacking, hydrogen bonding, electrostatic interactions, and solvation.^20^ Flexible side chains and hydrogen-bonding groups are often introduced to improve aqueous solubility and direct supramolecular organization. Detailed structural studies indicate that peri-imide substituents can form lateral hydrogen bonds between adjacent molecules, while π stacking stabilizes dimeric units that propagate into extended one-dimensional twisted chains; lateral association of two such chains through slipped π-stacking can further generate J-type aggregates.^14,21,22^ Depending on the strength and directionality of these interactions, PBIs may undergo cooperative supramolecular polymerization, with distinct nucleation and elongation regimes, or isodesmic assembly governed by a single elongation constant.^8,14^ Solvent–solute interactions are also central to this process, as shown by entropy-driven aggregation and recent atomistic simulations demonstrating that water can shape aggregate geometry and bias self-assembly pathways toward cooperative or anticooperative behavior.^14,20,23–25^

Despite extensive thermodynamic, spectroscopic, and structural studies, the solution kinetics of PBI self-assembly remain comparatively underexplored. This is a critical gap, particularly for biological applications such as cell staining, where binding, localization, and aggregate formation occur on experimentally relevant time scales. Kinetic studies also provide a necessary bridge between atomistic simulations and equilibrium thermodynamics. Historically, salt-induced aggregation of fluorescent dyes played a central role in the development of molecular exciton theory, and kinetic investigations on cyanine dyes have revealed sigmoidal aggregation profiles incompatible with simple diffusion-limited association.^26,27^ In contrast, analogous kinetic studies on PBI dyes remain scarce, partly because of their strong aggregation propensity in aqueous and physiological media.^7,22^

Here, we investigate the kinetics of salt-induced self-assembly of two representative PBI derivatives: the positively charged bis-spermine adduct PBI-S and the negatively charged tetracarboxylic acid derivative (PTC), whose detailed chemical structures, synthesis and NMR spectra are reported in the Supporting Information (Figures S1–S3). PBI-S has previously been studied as a potential DNA quadruplex fluorescent probe and telomerase inhibitor in cancer cells,^28–31^ and its aqueous and solid-state behavior has been characterized.^7,32^ AFM measurements on spin-coated films from aqueous PBI-S solutions revealed globular nanostructures 3–6 nm long, with a minimum diameter of 3.6 nm, consistent with the monomer longitudinal axis.^7^ Molecular dynamics simulations further showed a clear segregation between the rigid hydrophobic perylene core and the flexible hydrophilic spermine shell, with modeled aggregates displaying an overall diameter of approximately 4.3 nm and an inner π-stacked core of approximately 1.5 nm.

Ion-specific effects and ionic-strength screening are well-established in the aqueous aggregation of charged π-conjugated dyes, including PDI/PBI derivatives.^33^ In this work, we focus on NaCl in phosphate buffer to isolate the role of ionic strength under controlled pH while maintaining conditions that approximate physiological media. This choice is consistent with previous studies on water-soluble PDI/PBI systems, where phosphate buffer and NaCl were used to modulate aggregation equilibria and supramolecular assembly.^34^

Stopped-flow fluorescence experiments were used to monitor PBI-S aggregation at pH 7.2 and 25 °C as a function of NaCl concentration. Measurements were performed over 2–32 μM PBI-S and 20–320 mM NaCl after mixing, under low-optical-density conditions to minimize inner-filter effects and maintain an approximately linear fluorescence response. Representative traces are shown in Figure 1. Under the present 1 cm optical-path setup, concentrations above 40 μM could not be reliably investigated. No measurable dimer dissociation was observed upon 1:2 dilution in the 0.1–10 s time window, even in the absence of NaCl. The traces showed an initial fluorescence increase over 0–5 s, followed by a slower decay at longer times. The stopped-flow traces that showed the fluorescence increases were fitted with a monoexponential function to obtain an apparent relaxation rate *k*_obs_, allowing comparison across conditions (Figure 1B and Figure 2). A detailed discussion of the long-time traces shown in Figure 3 is provided below. As shown for 16 μM PBI-S in Figure 1A, *k*_obs_ increased only moderately from 0.36 ± 0.06 s^–1^ at 20 mM NaCl to 0.66 ± 0.05 s^–1^ at 320 mM NaCl, whereas the amplitudes increased by about 2 orders of magnitude. The extracted rates are consistent with a pseudo-first-order process, yielding a second-order rate constant of 7.4 × 10^4^ M^–1^ s^–1^, assigned to salt-independent PBI-S dimer formation, or nucleation, *k*_n_^+^.

**Figure 1:**
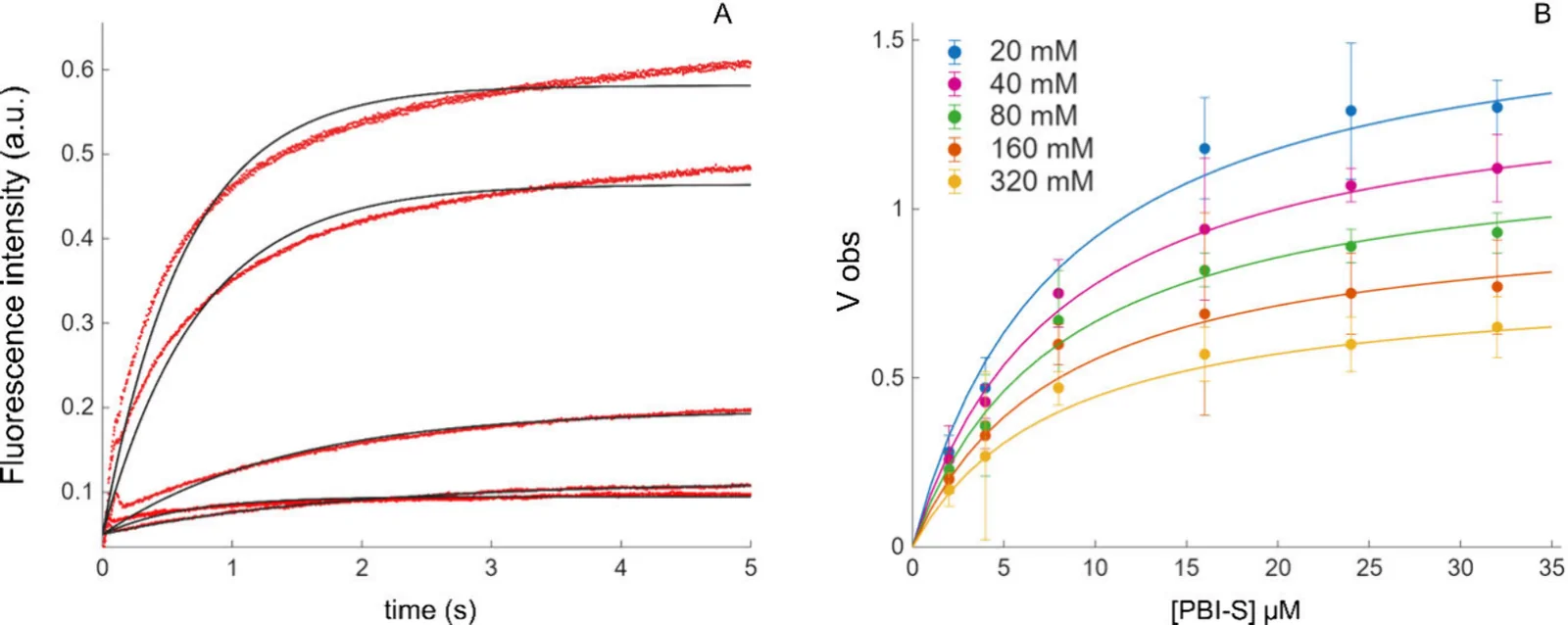
Time courses of PBI-S polymerization as a function of NaCl concentration. (A) Fluorescence stopped-flow measurements were carried out by mixing a 16 μM PBI-S solution (in 10 mM phosphate buffer pH 7.2) with increasing NaCl concentrations (from bottom to top: 20, 40, 80, 160, and 320 mM, in the same buffer). The excitation wavelength was set at λ_exc_ = 530 nm. Whole fluorescence signal was acquired after a passband filter at 545 nm (see also Figure S4). Experimental curves in red, single-exponential fitting curves in black. (B) Michaelis–Menten plot of the observed rates. The observed rates, calculated from data in panel (A), are plotted against PBI-S concentrations. Experimental points were fitted according to the simple Michaelis–Menten hyperbola from which a single *K*_M_ of 9.9 μM was obtained (see eq 1).

**Figure 2:**
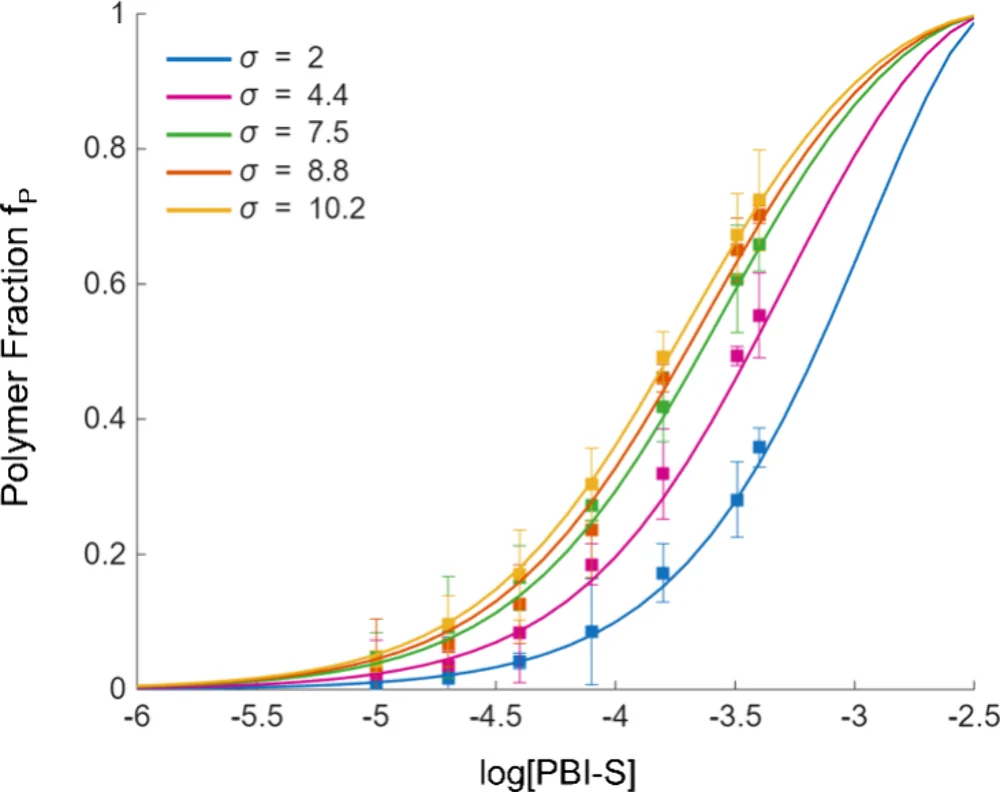
Observed amplitudes for NaCl induced PBI-S polymerization under stationary state. The amplitudes relative to the time courses depicted in Figure 1A, are plotted as a function of PBI-S concentrations. Typical “stationary state” binding isotherms are obtained whose profiles are satisfactorily accounted for by the *K*_2_–*K* thermodynamic model as described in the text and Supporting Information by assuming *K*_2_ = 1/*K*_M_.

**Figure 3:**
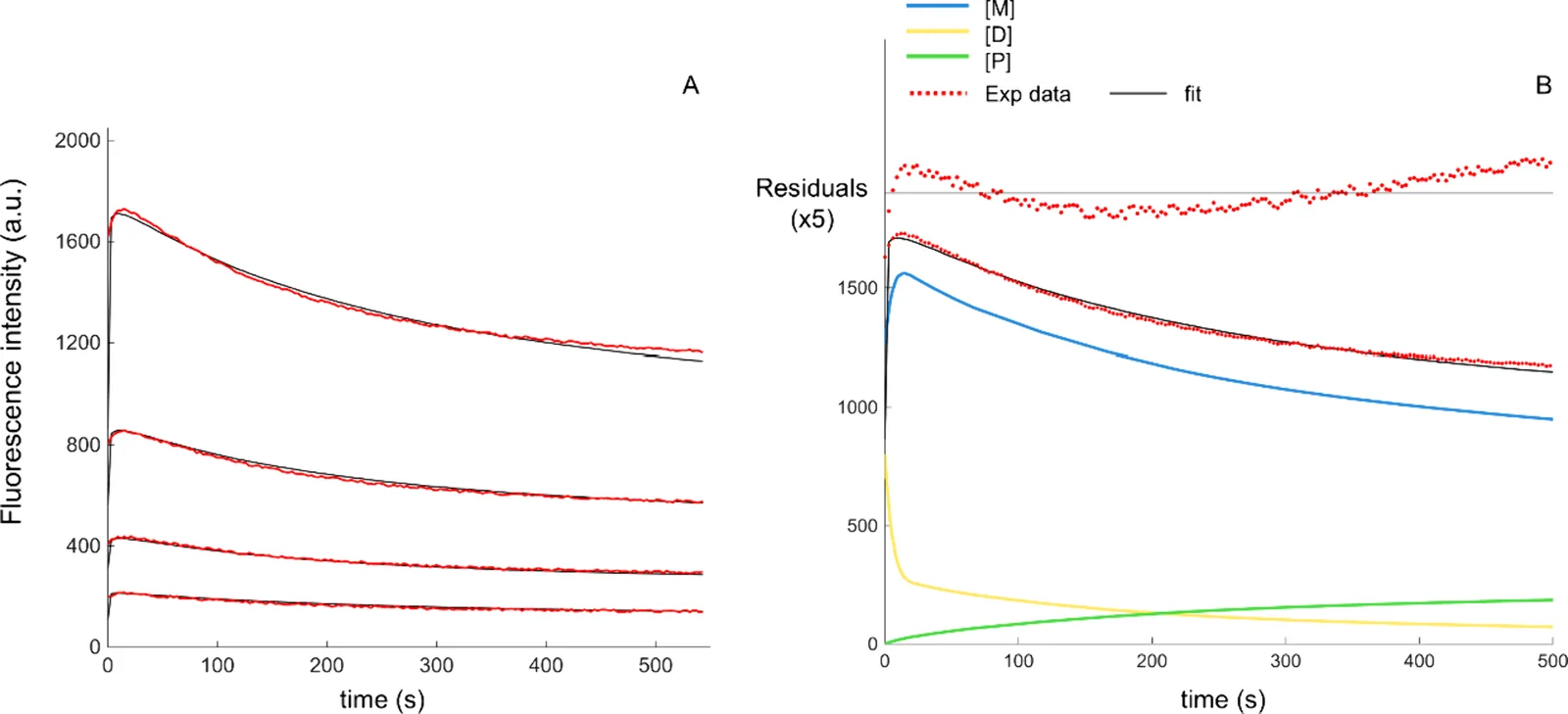
Experimental fluorescent kinetic traces (panels (A) and (B)) are shown in red, while numerical ODE fitting curves are in black. Temperature was set at *T* = 25 °C. The excitation and emission wavelengths were λ_exc_ = 530 nm and λ_emiss_ = 560 nm (see also Figure S4). Fluorescence spectrofluorometer measurements (5 nm excitation and emission slits) were carried out by mixing a 16 μM PBI-S solution (in 10 mM phosphate buffer pH 7.2) with increasing NaCl concentrations (from bottom to top: 10, 20, 40, 80 mM, in the same buffer). In panel (B), the relative fluorescence emission amplitudes of monomer, dimer and polymers, as obtained from ODE fitting to the data at 80 mM NaCl and 16 μM PBI-S solution (top curve of panel A) are shown. Top inset shows residuals (data points – fitting curve) magnified by 5. Five parameters were allowed to float (see the Supporting Information), namely, the three amplitudes: *A*_1_ = 765 ± 76 for the monomer (M), *A*_2_ = 1781 ± 109 for the dimer (M_2_), and *A*_3_ = 685 ± 99 for the polymer (P), together with the two constants *k*_e_^+^ and *k*_e_^–^ (values indicated in the text). The robustness of the fit is estimated R^2^ = 0.879.

A complete matrix of rates and amplitudes was thus obtained as a function of both PBI-S (from 2 to 32 μM) and NaCl (from 20 to 320 mM) concentrations. The observed rates at different NaCl concentrations, plotted as a function of PBI-S concentrations (see Figure 1B), immediately provide a straightforward phenomenological interpretation, yielding hyperbolic saturation profiles typical of a Michaelis–Menten-like behavior (that is, valid only in the presence of excess monomer species). Accordingly, a rate-limiting *V*_MAX_ (*v*_obs_ at asymptotes) can be singled out at high fluorophore concentrations, whose value is inversely dependent on salt concentration (from 0.5 to 1.75 s^–1^) and a *K*_M_ value of 9.9 μM, independent of NaCl concentrations. So far, the whole picture at stationary state, can be described by eq 1, according to the simplest stationary state model (where M = monomer, M_2_ = dimer, P = polymer) with values and statistical goodness-of-fit parameters listed in Table [Table tblI].

$$\begin{array}{c} M+M\overset{k_{n}^{+}}{\underset{k_{n}^{-}}{⇌}}M_{2}\overset{V_{\mathrm{MAX}}}{\to }P\\ v_{\mathrm{obs}}=V_{\mathrm{MAX}}\frac{\left[M\right]}{K_{M}+\left[M\right]} \end{array}$$ 1

**1 tblI:** Stationary State Parameters Obtained from the Curve Fitting of Figure 1B

Parameter	Value	Units	Notes
*K*_2_ = 1/*K*_M_	(1.00 ± 0.11) × 10^5^	M^–1^	Nucleation equilibrium constant (R^2^ = 0.983)
*k*_*n*_^+^	(7.40 ± 0.08) × 10^4^	M^–1^ s^–1^	Association rate constant (R^2^ = 0.988)
*k*_*n*_^–^	0.73 ± 0.08	s^–1^	Calculated as *k*_*n*_^+^/*K*_2_
*V*_MAX_ (20 mM)	1.75	arb. units	Monotonic decrease with salt concentration
*V*_MAX_ (40 mM)	1.42	arb. units	
*V*_MAX_ (80 mM)	0.98	arb. units	
*V*_MAX_ (160 mM)	0.73	arb. units	
*V*_MAX_ (320 mM)	0.54	arb. units	

In this scenario, the dimer behaves as a transient, kinetically competent nucleus whose steady-state concentration leads to an effective polymerization rate that is first-order in M_2_ and displays saturation with increasing monomer concentration. This kinetic behavior is fundamentally distinct from a mechanism in which polymer growth occurs exclusively through bimolecular encounters between dimers, M + M ⇌ M_2_, followed by M_2_ + M_2_ → M_4_. In the latter case, polymer formation is second-order in the dimer concentration, resulting in a strongly nonlinear dependence of the overall rate on monomer concentration (pronounced lag phases) and sigmoidal growth kinetics, characteristic of cooperative nucleation processes. The absence of saturation behavior and the enhanced sensitivity to monomer concentration in the M_2_ + M_2_ → M_4_ pathway therefore provide clear kinetic criteria to discriminate between these two mechanistic regimes.

In parallel, the observed amplitudes, as obtained from the end points of the kinetic time courses of Figure 1A, can be more conveniently plotted against PBI-S concentrations where the whole grid of data shapes a set of stationary state titration profiles at each given salt concentration (Figure 2). Data were fitted to the simplest *K*_2_–K model (see the Supporting Information for complete derivation) using the *K*_2_ value obtained from Table [Table tblI].

M+M⇌K2M2M2+M⇌KM3M3+M⇌KM4...

The total concentration (*c*) is given as *c* = *c*_M_ + 2*c*_M_2__ + 3*c*_M_3__ + ... + *nc*_M_*n*__.

If σ = *K*_2_/*K* and by setting *x* = *K*_cM_, the observable fraction of polymeric species (including dimers) (represented as *f*_p_) is defined as shown in eq 2:

2 $$f_{p}\left(x\right)=\frac{\sigma \cdot \frac{2x-x^{2}}{{\left(1-x\right)}^{2}}}{1+\sigma \cdot \frac{2x-x^{2}}{{\left(1-x\right)}^{2}}}$$

As shown in Figure 2, the PBI-S binding curves, recorded at fixed NaCl concentrations, are well-described by a simple anticooperative *K*_2_–*K* model. Within this framework, the nucleation constant *K*_2_, obtained as 1/*K*_M_ from Figure 1B, is independent of salt concentration. Conversely, the fitted parameter σ = *K*_2_/*K* increases almost linearly with NaCl concentration, corresponding to a progressive decrease in the elongation constant *K*, from 4.8 × 10^4^ to 9.4 × 10^3^ M^–1^. The binding isotherms were fitted independently at each NaCl concentration, rather than by global fitting; therefore, the reported *K* values represent separately optimized elongation constants. Despite the simplified stationary-state approximation, the fits were consistently robust, with individual R^2^ values of approximately 0.95–0.99 and an average R^2^ of about 0.97.

This analysis provides a quantitative description of the early stationary-state regime, approximately spanning 5–20 s, and allows reliable extraction of dimerization-related parameters. However, it does not describe the full kinetic evolution of the system toward equilibrium. In particular, although the inverse dependence of both *V*_MAX_ and *K* on salt concentration is consistent with a salt-perturbed elongation equilibrium, it does not provide direct access to the elementary rate constants *k*_e_^+^ and *k*_e_^–^ governing polymer growth and dissociation. Therefore, a complete kinetic description requires a time-dependent model based on the full set of differential equations.

To address this limitation, we extended the analysis monitoring the fluorescence evolution over 100 s, performed on a manual stopped-flow spectrofluorometer (Figure 3). These measurements capture the complete kinetic trajectory: an initial fluorescence increase, assigned to the formation of emissive dimers, followed by a gradual decay associated with the accumulation of polymeric species. Although these polymers remain fluorescent, their emission intensity is lower than that of the dimer, indicating substantial aggregation-induced quenching.

The experimental curves (Figure 3A) and their ODE-based fits (Figure 3B) support the use of a truncated kinetic *K*_2_–*K* model:

$$M+M\overset{k_{n}^{+}}{\underset{k_{n}^{-}}{⇌}}M_{2}+M\overset{k_{e}^{+}}{\underset{k_{e}^{-}}{⇌}}M_{3}...$$

Numerical solutions of the ODE system required careful initialization due to stiffness introduced by the fast nucleation and slow elongation kinetics. Accordingly, small seed values for the dimer species (for example, *c*_M2_(0) = 1 μM) were introduced, which were consistent with mechanistic expectations and previously reported equilibrium data in plain water.^8,35^ Under these conditions, the *k*_e_^+^ value was assessed as 42 ± 15 M^–1^ s^–1^, whereas *k*_e_^–^ was dependent on the salt concentration and varies between (1.5 ± 0.4) × 10^–2^ and (6.2 ± 2.7) × 10^–4^ s^–1^ at 20 and 320 mM NaCl concentrations, respectively. The estimates of the slow rate constants are affected by significant errors (±30%; R^2^ = 0.879) due to the contribution of the PBI-S aggregates precipitation process occurring within tens of minutes after salt mixing.

The observed kinetic profile can be interpreted in light of recent nanosecond-scale atomistic simulations on a structurally related perylene bisimide-diamine derivative in water.^24^ These simulations showed that aggregation is intrinsically anticooperative and proceeds through emissive dimers as the main structural units. Higher-order stacks form stepwise, but their growth is progressively limited by cumulative enthalpic penalties and decreasing entropic gains from water release. As stack size increases, both contributions eventually plateau, producing a size-independent free-energy change that limits further extension. The average π–π stacking distance of 3.7–3.9 Å, together with restricted rotational and translational motions in even-numbered stacks, further supports the dimer as a privileged kinetic unit. This interpretation agrees with our second time-regime kinetic data, where dimer formation precedes and influences elongation. However, a mechanism in which dimers directly act as the main elongating units, for example through dimer-to-tetramer growth, is not supported by the stationary-state analysis in Figure 1.

In this integrated view, the kinetic data and molecular dynamics simulations together suggest that dimer formation is both kinetically dominant and structurally privileged. Polymer growth, although thermodynamically allowed, is modulated by solvation dynamics and stacking frustration, with salt perturbations selectively stabilizing intermediate aggregates. NaCl may modulate polymer stability by reducing electrostatic repulsion and by promoting specific interactions between chloride ions and protonated spermine groups. Since side chains remain hydrated while solvation of the aromatic cores and imide carbonyls decreases with aggregate length, water expulsion is expected to occur through a stepwise, partially hydrated pathway, reinforcing anticooperative assembly.

In this framework, chloride binding may stabilize intermonomer contacts during elongation and lower the polymer dissociation rate, *k*_e_^–^. Similar effects have been reported for anionic PDI derivatives, where anion binding enhances stacking persistence,^13,36,37^ and aggregate-based anion sensing has recently been linked to anion-driven polymerization.^38^ Upon binding of anions to cationic sites along the polymer chain, polymerization is expected to decrease once all available sites become saturated.^39^ As in the present study, kinetic barriers due to precipitation process may enter into the mechanism, thereby increasing its overall complexity. Taken together, these salt-induced effects suggest that NaCl not only modulates the thermodynamic equilibrium of PBI-S assembly but also acts kinetically by stabilizing intermediate aggregates and lowering dissociation probabilities. This dual action reinforces the mechanistic picture of entropically driven, solvation-modulated self-assembly and opens new perspectives for rational control of π-stacked supramolecular polymers under diverse solvent conditions.

The spectroscopic properties of PBI-S and PTC in aqueous media were examined by considering the contributions of monomeric, dimeric, and higher-order aggregated species, with the aim of unambiguously defining their characteristic absorption and fluorescence line shapes. Because vibronic structure plays a central role in interpreting these spectra, resonant Raman spectroscopy has, in principle, the capabilities to identifying the vibrational modes that govern the observed progressions. As demonstrated by Greiner and Sundholm for perylene,^40^ the vibrational frequencies of the S0­(Ag) and S1­(B3u) states differ by less than 15 cm^–1^, suggesting that analogous behavior may be expected for PBI derivatives. Raman data on PBIs, however, remains scarce, primarily due to the difficulty of acquiring reliable spectra in the presence of strong fluorescence. To circumvent this limitation, stimulated Raman scattering (SRS) was performed under red-shifted (preresonant) excitation relative to the visible absorption maximum (see the Supporting Information). SRS arises from a third-order nonlinear optical interaction between a femtosecond broadband probe pulse (PP) and a narrowband picosecond Raman pump (RP). Briefly, Raman-active modes whose energy matches the energy difference between the RP, and the PP spectral components are coherently stimulated,^41,42^ resulting in a modulation of the macroscopic polarizability of the medium. This, in turn, gives rise to Raman features on top of the probe spectrum, which can be isolated via the Raman gain, defined as

$$\mathrm{RG}\left(\omega \right)=\frac{I_{\mathrm{ON}}\left(\omega \right)-I_{\mathrm{OFF}}\left(\omega \right)}{I_{\mathrm{OFF}}\left(\omega \right)}$$

where *I*_ON_(ω) and *I*_OFF_(ω) are the PP spectrum in the presence/absence of the RP, respectively. When the RP is tuned into resonance with the sample absorption, the Raman response is selectively enhanced.^41,43^ Under both resonant and nonresonant conditions, the red-shifted SRS corresponds to a Stokes-side spontaneous Raman spectrum. A key advantage of SRS over spontaneous Raman spectroscopy, however, is the effective suppression of fluorescence background. Under resonant excitation, fluorescence represents a dominant contribution in spontaneous Raman measurements, as both are emitted without directional selectivity. In striking contrast, the SRS signal arises from a coherent light–matter interaction and is emitted collinearly with the probe beam, enabling selective detection of the vibrational response.^44^ Vibrational assignments were made by analogy with the parent iso-symmetric perylene chromophore, whose Raman and vibronic structure has been extensively characterized in solution and in inert matrices.^40,45–47^ In perylene, only a limited set of fundamental modes (348, 543, 1098, 1293, 1395, and 1601 cm^–1^) accounts for the majority of vibronic intensity in the S1­(B3u) ← S0­(Ag) electronic transition (see Table S1 in the Supporting Information), providing a useful reference framework for the interpretation of PBI derivatives.

To achieve optimal signal-to-noise ratios, we employed a relatively high absorbance (>1 AU) in a thin optical cell (1 mm), constraining PBI-S concentrations to >50 μM. At such concentrations, purely monomeric populations cannot be maintained in water, in agreement with previous absorption studies on PBI-S.^7^ Consequently, high-absorbance spectra representative of the monomeric species were obtained in trifluoroacetic acid (TFA), and systematic dilutions of PBI-S/TFA solutions into water were carried out to monitor the spectral signatures associated with aggregation.

Thus, in Figure 4A and 4B, the spectrum in TFA is reported, where PBI-S is predominantly in its monomeric species together with the spectrum of PBI-S in a 50% mixture of water/TFA, where non-negligible aggregate formation is observed (decrease in the ratio 0–0 to 0–1 absorption peaks of PBI-S; also see Figure 5). Spectra were also acquired in water, where dimeric species are the predominant form, and at high salt concentration, where polymeric species predominate. The same setup was used to measure stimulated Raman spectra of PTC, that is consistently monomeric at all concentrations used, independent of the presence of salt (Figures 4C and 4D).

**Figure 4:**
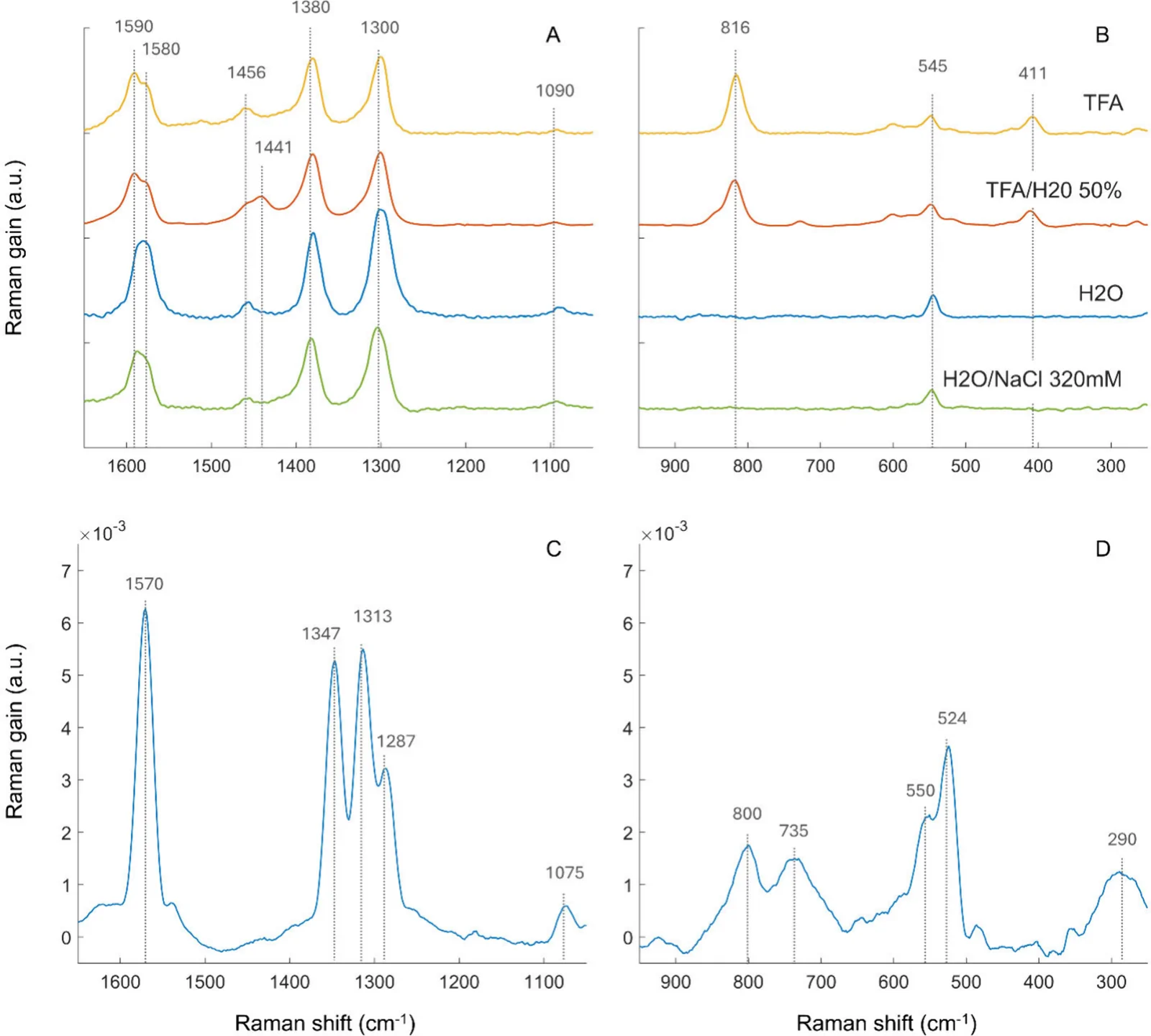
Stimulated Raman emission spectra of the two perylene-based compounds. Panels A and B show spectra of PBI-S under different solvent conditions. Samples concentrations were about 290 μM. Spectra are shown in the high-frequency (panel A) and low-frequency (panel B) ranges. Gray dashed lines indicate the most relevant peaks detected. Off-resonant SRS (580 nm) was induced with Ti:sapphire laser (800 nm, 50 fs, 1 kHz). The probe was a white-light continuum generated in a 3 mm sapphire crystal. Resonant SRS (448 nm) was induced with Yb:KGW laser (1030 nm, 190 fs, 10 kHz). The probe was generated from the second harmonic in a 6 mm sapphire crystal. Panels C and D show SRS of PTC in water (compound concentration was 600 μM). Spectrum is shown in the high-frequency (panel C) and low-frequency (panel D) ranges. Gray dashed lines indicate the most relevant peaks detected. The experimental setup is the same as that used in the experiments of PBI-S. Off-resonant SRS (580 nm): Ti:sapphire laser (800 nm, 50 fs, 1 kHz). Resonant SRS (448 nm): Yb:KGW laser (1030 nm, 190 fs, 10 kHz). In both setups, the Raman pump was spectrally compressed to few-ps pulses with ∼7–10 cm^–1^ bandwidth and ∼1 μJ energy. Probe was generated from the second harmonic in a 6-mm sapphire crystal.

**Figure 5:**
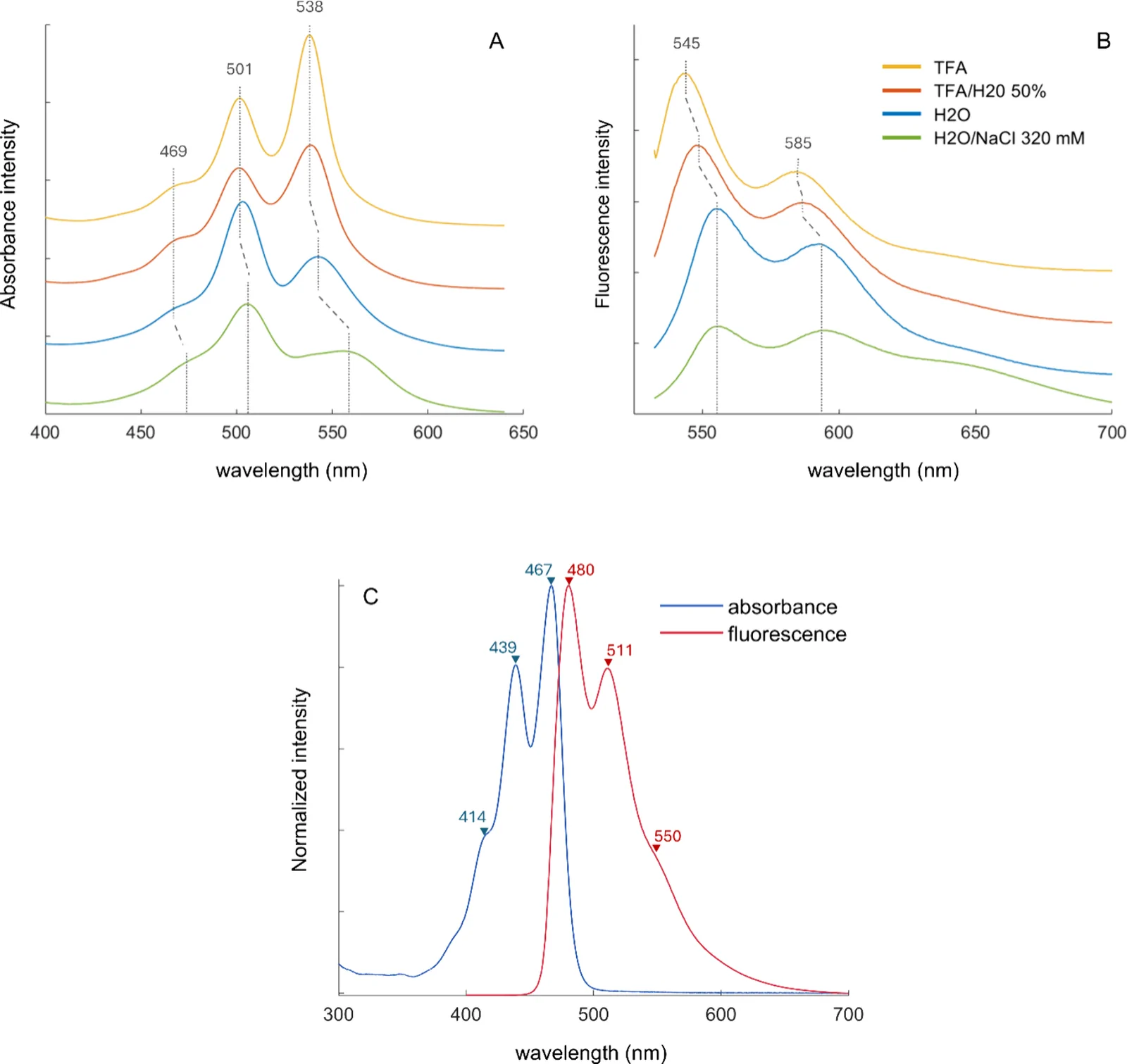
Absorbance and fluorescence spectra of PBI-S (panels A and B) and PTC (panel C). PBI-S was dissolved in TFA and/or water. Spectra of 1.375 × 10^–5^ M PBI-S were acquired in 1 cm optical path. Fluorescence emission spectra were obtained with λ_exc_ of 467 nm (5 nm slits) at 20 °C. PTC was dissolved in water and its spectra are representative of the monomeric state at room temperature. No spectral changes are observed upon concentration ranges between 1 × 10^–7^ and 1 × 10^–2^ M. Molar absorptivity, assessed by standard gravimetric measurements, indicated a value of 17 450 M^–1^ cm^–1^.

The Raman peak assignments summarized in Table S1 follow from the spectroscopic considerations outlined in the preceding sections, with particular reference to stimulated Raman spectroscopy. Comparable assignments were reported in 2000 by Scholz et al. for PTC dianhydride in epitaxial films^16^ and in 2007 by Clark et al., based on theoretical calculations,^48^ both in agreement with the present data.

The intense features observed around 1590–1580 cm^–1^ in PBI-S and 1570 cm^–1^ in PTC correspond to C–C stretching along the long axis of the perylene core, consistent with the dominant high-frequency normal modes previously identified in perylene (74 and 73 in the work of Ong et al.^47^). These features illustrate the “perylene-like” vibrational signature carried over into PBI-S and PTC, which is consistent with the discussion that PBI derivatives retain a vibrational framework closely resembling the parent molecule. Their conservation across PBI-S and PTC confirms that these modes represent fundamental components of the vibronic progression described earlier. The 1456 cm^–1^ band and the 1380 cm^–1^ feature fall in the midfrequency region associated with in-plane C–C–H bending, C–H in-plane deformation, or vinyl stretching along the long molecular axis. These frequencies align closely with perylene modes 69 and 66 reported by Ong et al.,^47^ again reinforcing the analogy emphasized in the text. Importantly, the 1380 cm^–1^ mode, one of the key vibrations contributing to vibronic structure in perylene, is clearly observed in both PBI-S and PTC, indicating that SRS successfully isolates this signature even under conditions of high fluorescence. While PBI-S exhibits a single 1300 cm^–1^ mode analogous to perylene mode 60, PTC shows a split band (1313, 1287 cm^–1^), possibly indicating the coexistence of distinct molecular conformations.

The peaks at 1090/1075 cm^–1^, 816/800 cm^–1^, and associated weak features correspond to C–C bending, in-plane C–H deformations, and out-of-plane aromatic bending, in agreement with perylene fundamentals 48, 33–34, and 32. Low-frequency skeletal torsions (550–290 cm^–1^) are considered as sensitive indicators of aggregation and molecular interactions. The 545 cm^–1^ band in PBI-S and the split 550/524 cm^–1^ modes in PTC correspond to biphenyl bending and mixed C–C–C skeletal motions, matching perylene modes 20 and 16–19. This last contribution is, however, also present in the aggregated SR spectra of Figure 4 as a single peak and is consistently the only low frequency feature of vibrational spectrum of the aggregates. As a working hypothesis, we may consider that the features between 525 and 550 cm^–1^ are composite (see Table S1) and the out-of-plane components are quenched upon aggregation whereas the in-plane C–C biphenyl stretching is, in principle, unaffected. Likewise, the lowest-frequency skeletal torsions (411 and 290 cm^–1^) appear as weak monomer-derived features, aligning with perylene modes 11 and 8.

Taken together, these observations show that low-frequency vibrations (<800 cm^–1^) appear as strong or medium-intensity features in the monomeric forms (PBI-S in TFA, PTC in water) but become markedly suppressed in the H-aggregated PBI-S species. This global attenuation of low-frequency activity in aggregated samples supports at least two complementary interpretations: (i) structural (packing) constraints in the cofacial H-aggregate restrict the amplitude of out-of-plane modes, effectively quenching torsional motion, (ii) exciton-induced vibrational compression (“exciton pressure”) selectively stabilizes or suppresses low-frequency ground-state modes within the aggregate.

These two perspectives could be merged according to the exciton-vibrational coupling model of Spano and Silvestri^49^ and the key experimental observation of Ghosh et al.,^50^ who demonstrated how exciton-vibration coupling in planar aromatic systems can be rationalized based on vibrational frequencies of relevant modes as compared to the type of electronic transition, that is charge-transfer (CT) band vs π–π*. In systems with strong HOMO–LUMO overlap (namely, π–π*) and dense electronic charge near high-frequency nuclear motions, electron–phonon coupling is dominated by high-frequency modes (large Huang–Rhys (HR) factors) and fast excitation decay rates. Conversely, low-frequency torsional modes (<800 cm^–1^), which are spatially more delocalized, are only weakly coupled to the electronic π–π* transition and, thus, are more sensitive to excitonic compression or packing constraints and consequently characterized by slower decay rates. The reverse is observed in the case of CT bands with highly delocalized electronic transitions.^50,51^

The behavior observed for PBI-S H-aggregates aligns precisely with this mechanistic picture, and the strong low-frequency signals seen in monomeric PBI-S and PTC further validate this interpretation. A more complete assignment of these low-frequency modes on an experimental basis would, however, be necessary to fully disentangle structural and excitonic contributions.

The following dataset defines the absorption and fluorescence signatures of monomeric and aggregated PBI-S and PTC in aqueous solution, used here as model PBI chromophores. Rather than revisiting previously reported equilibrium titrations,^7^ we first identify the spectral features of the purely monomeric species. PTC shows no detectable spectral changes over 5 orders of magnitude in concentration or upon addition of NaCl up to 1 M (data not shown). This rules out compensation between coexisting bright and dark excitonic species, or a “null spectrum”,^51^ and indicates that PTC behaves as a strictly monomeric chromophore. Accordingly, PTC provides a reliable reference for a Franck–Condon (FC) vibronic line shape (Figure 5).

In contrast, PBI-S shows the expected aggregation signatures (Figure 5). Its absorption spectrum agrees with literature data and displays a reduced 0–0/0–1 intensity ratio, consistent with H-type aggregation. Whereas PTC is described by a FC progression dominated by a high-frequency mode at 1340 ± 60 cm^–1^, with *S* ≈ 0.65, PBI-S dimers show non-FC vibronic line shapes and an approximately halved effective HR factor of ∼ 0.32. These features identify the aggregates as vibronic excitons with possible charge-transfer exciton admixture,^51^ although for Herzberg–Teller (HT) or Franck–Condon/Herzberg–Teller interference, coupling cannot be excluded.^52,53^

The addition of 320 mM NaCl further broadens the fluorescence emission on the red side, consistent with higher-order aggregate formation and possible excimer contributions.^54^ All measurements were performed within the time window used for SRS. Transient fluorescence experiments show that predominantly dimeric species are more emissive than either monomers or larger aggregates. This behavior is consistent with a transition from monomeric FC vibronic progressions to excitonically coupled dimers, where oscillator strength is redistributed and vibronic intensity ratios are modified.^52^ In this framework, electronic-state mixing alters the effective excitonic coupling without necessarily requiring an explicitly coordinate-dependent, HT-type transition dipole. Exciton delocalization within H-dimers may reduce the effective HR factor by distributing electronic reorganization energy over multiple chromophores, thereby weakening the apparent electron–phonon coupling while preserving rapid intraband relaxation.^55^ This interpretation is consistent with theoretical simulations of PDI dimers and with the experimentally observed fluorescence enhancement and asymmetric suppression of low-frequency Raman modes.

More generally, PBI dimer spectroscopy has been described using models ranging from the Holstein exciton–vibrational framework to first-principles and quantum-vibronic approaches that include conformational disorder, solvation, local excitations, and charge-transfer states.^15–17^ These models aim to rationalize aggregation-induced quenching (AIQ) and aggregation-induced emission (AIE) in multiparticle assemblies.^56,57^ Further progress will require stronger experimental constraints, particularly through time-resolved SRS combined with cryogenic absorption and emission measurements, which could help isolate discrete oligomeric species and correlate structural heterogeneity, vibronic dynamics, and macroscopic photophysical behavior.

This study elucidates the kinetics of self-assembly and spectroscopic behavior of perylene bisimide derivatives in aqueous environments, highlighting the interplay among NaCl concentration, aggregation kinetics, and vibronic coupling. PTC flow kinetic analyses under stationary state demonstrate that PBI-S aggregation follows an anticooperative *K*_2_–*K* model, where dimer formation acts as the salt-independent nucleation step, while elongation and polymer stability are strongly modulated by ionic strength. Increasing NaCl concentration enhances polymer persistence by suppressing monomer release, leading to larger and less soluble aggregates. In this context, physiological divalent cations may also be relevant within the intracellular environment, although their specific role lies beyond the scope of the present study and will be addressed in future investigations. Complementary stimulated Raman spectroscopy reveals that two dominant, spectrally distinct species—that is, monomer and dimer/polymer—account for the observed salt-dependent spectral variations. The “desertification” of low-frequency, mostly out-of-plane, vibrational modes in aggregated states indicates restricted molecular flexibility and decoupled vibrations of these modes within π-stacked assemblies. As yet, in absorption and fluorescence spectra, dimeric and polymeric species contributions are distinct and, as also observed previously, are accounted for by exciton induced “non-Franck-Condon” vibronic coupling.^18^ Overall, this integrated kinetic-spectroscopic approach provides a coherent mechanistic framework for interpreting PBI aggregation under physiological conditions and underscores the relevance of controlled ionic environments in the rational design of PBI-based fluorescent probes and supramolecular architectures.

## Supplementary Material



## Notes

Chemical structures and synthesis protocols of PBI-S and PTC; NMR spectra of PBI-S and PTC; experimental setup for fluorescence measurements; experimental settings for stopped-flow, spectrofluorimetric, and stimulated Raman spectroscopy measurements; steady-state and time-dependent kinetic analyses; normal mode assignments of the stimulated Raman spectroscopy peaks of PBI-S and PTC.PDF)

## Abbreviations

PBI-S: spermine-functionalized perylene bisimide
PTC: perylene-3,4,9,10-tetracarboxylic diimide
PDI: perylene diimide
SRS: stimulated Raman scattering
PP: probe pulse
RP: Raman pump
ODE: ordinary differential equation
TFA: trifluoroacetic acid
CT: charge transfer
HR: Huang–Rhys
FC: Franck–Condon
HT: Herzberg–Teller
AIQ: aggregation-induced quenching
AIE: aggregation-induced emission
