# Supplementary material for: Electrolyte-Controlled Self-Assembly of Water-Soluble Perylene Bisimides: A Kinetic and Stimulated Raman Study
Source: J Phys Chem Lett. 2026 Aug 28;17(36):10378–87. doi: 10.1021/acs.jpclett.6c02220 (PMC13573606; doi:10.1021/acs.jpclett.6c02220)
Supplement: Supplementary file 1 [file jz6c02220_si_001.pdf]

Supplementary Information

Electrolyte-Controlled Self-Assembly of Water Soluble Perylene Bisimides: A Kinetic and Stimulated Raman Study

Miles Martinati*, Giovanni Batignani*, Gaia De Maria, Giancarlo Fabrizi, Yuri Gazzilli, Valeria Pennacchietti, Fang Yang, Alberto Boffi, Tullio Scopigno^, Roberta Piacentini

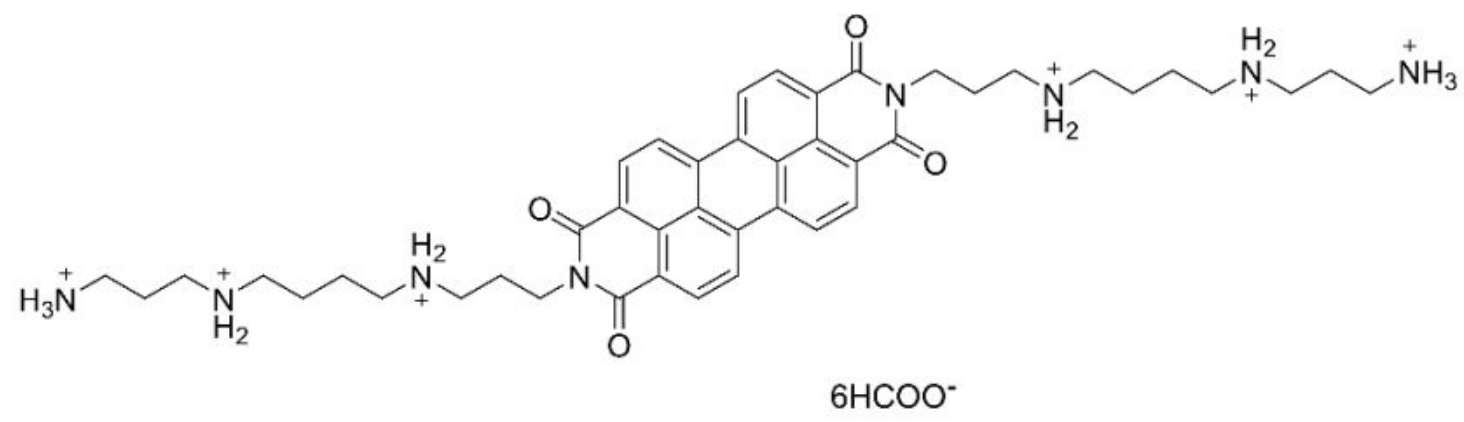

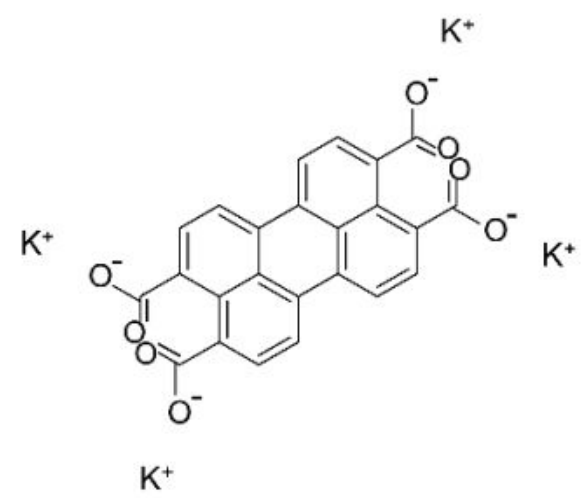

Figure S1. Chemical structure of PBI-S and PTC. Empirical formula of PBI-S is $[C_{50}H_{68}N_8O_{16}]^{6+}$·6HCOO$^-$. Molecular weight of cationic PBI-S is 1037.13 g/mol. Empirical formula of PTC is $C_{24}H_8O_8^{4-}$ · 4K$^+$. Molecular weight of anionic PTC is 424.3 g/mol.

Synthesis protocol of PBI-S: The perylene diimide PBI-S was synthesized following the procedure reported by Franceschin et al. (2008).[29] Briefly, perylene anhydride (1g) was reacted with a large molar excess of spermine hydrochloride (50-fold) to minimize undesired polymerization. The reaction was carried out in a DMA/dioxane mixture (40:60, v/v) under an argon atmosphere with continuous stirring, first at 100 °C for 24 h, and subsequently at 170 °C for 2 h. After completion, the solvents were removed by filtration. The resulting residue was dissolved in formic acid, precipitated as the corresponding salt, and washed repeatedly.

Synthesis protocol of PTC: In a 100 mL round-bottom flask with a magnetic stirring bar, PTCDA (250 mg, 0.637 mmol, 1 equiv.) and KOH (358 mg, 6.38 mmol, 10 equiv.) were added in 10 mL of H_2O and stirred at 90 °C for 2 hours. Subsequently, the reaction mixture was concentrated under reduced pressure, to yield 92% of potassium perylene-3,4,9,10-tetracarboxylate (340 mg).

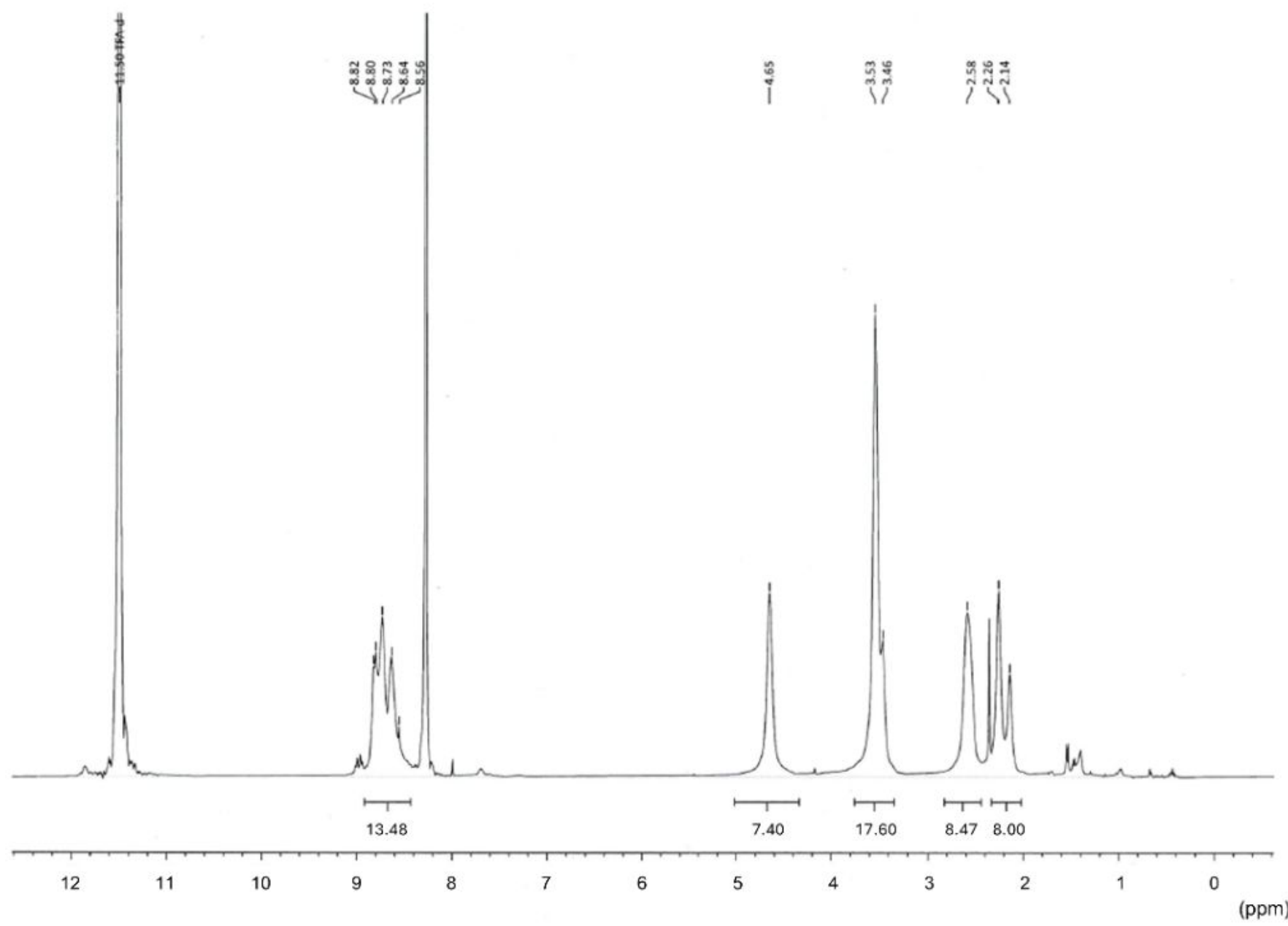

Figure S2. NMR spectrum of PBI-S (^{1}H NMR-spectrum, 400 MHz, d-TFA solvent).

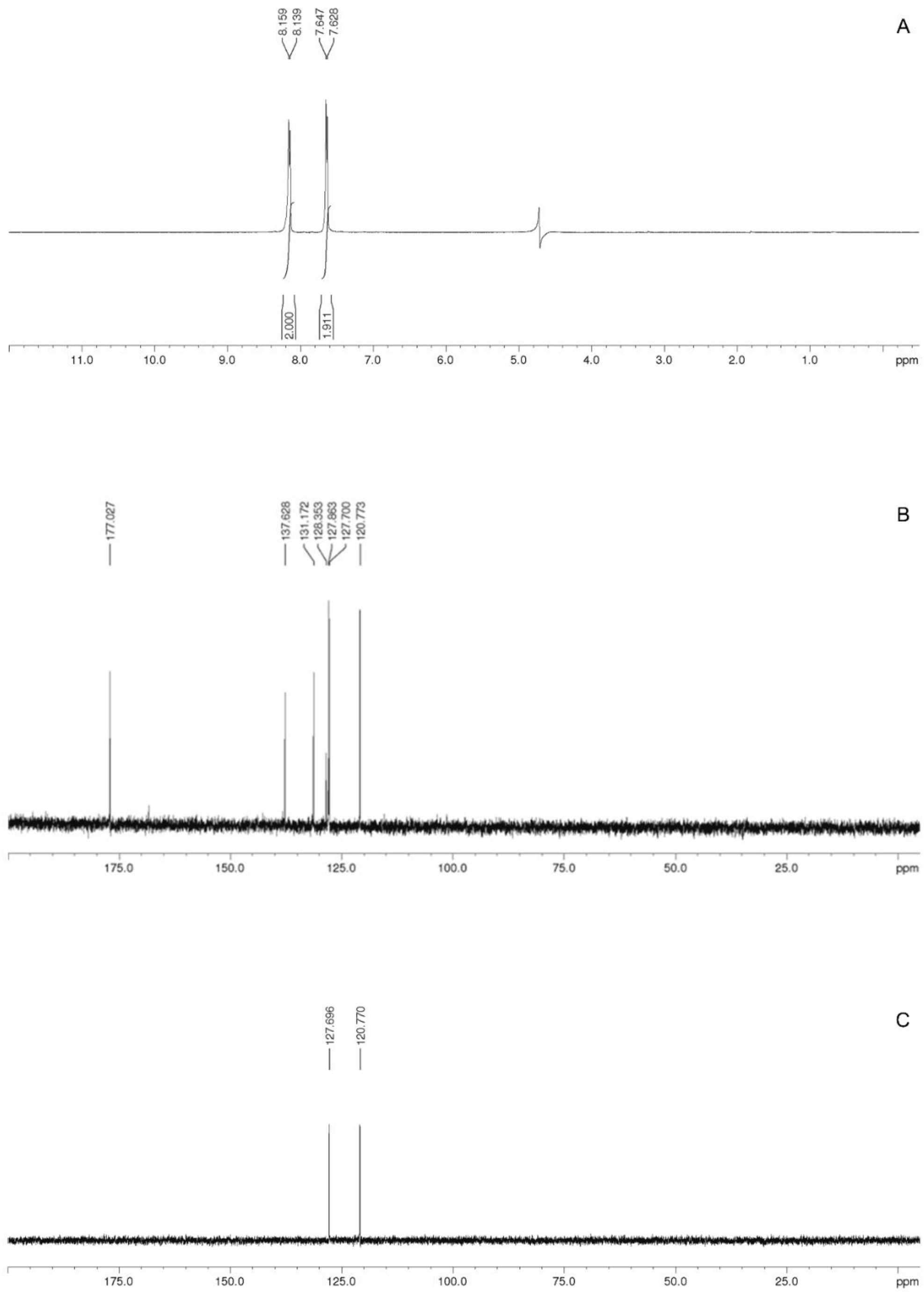

Figure S3. Panel A: ^{1}HNMR spectrum of PTC (400 MHz, D_2O with water suppression). Panel B: ^{13}CNMR spectrum of PTC (100.3 MHz, D_2O). Panel C: ^{13}CNMR DEPT 135 spectrum of PTC (100.3 MHz, D_2O). From NMR analysis: 92% yield; red solid; mp: dec. >300 °C; ^{1}H NMR (400.13 MHz) (D_2O): δ 8.14 (d, J = 7.8 Hz, 2H), 7.63 (d, J = 7.6 Hz, 2H); ^{13}C NMR (100.6 MHz) (D_2O): δ 177.0 (4C), 137.6 (2C), 131.1 (2C), 128.3 (2C), 127.8 (2C), 127.7 (2CH), 120.7 (2CH).

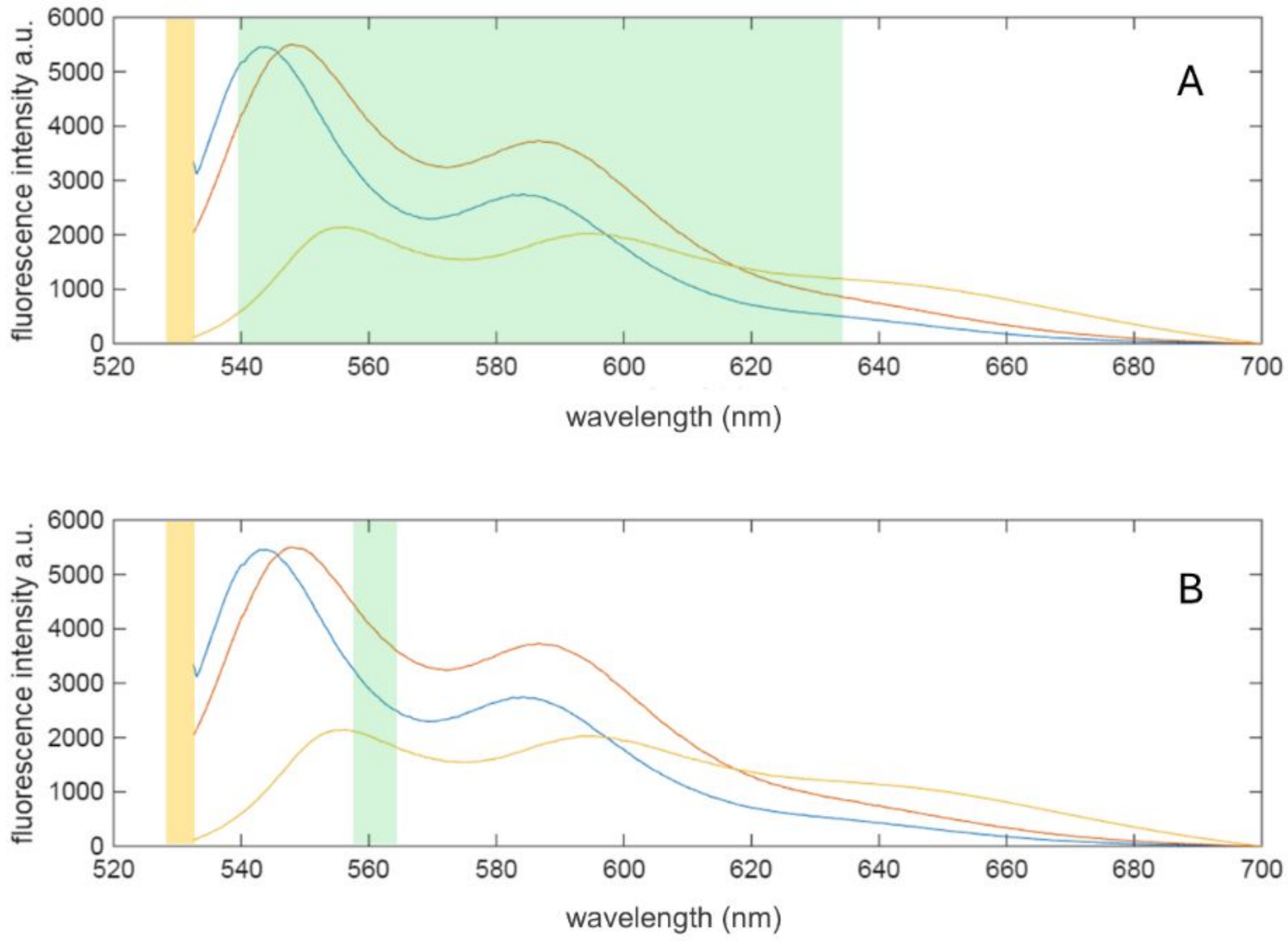

Figure S4. Experimental setup of PBI-S fluorescence emission. Spectra of the monomer (blue), dimer (red) and polymer species (yellow) are shown together with excitation bandwidth (orange shaded boxes) and emission bandwidth (green shaded boxes) relative to stopped flow experiments (panel A), and spectrofluorimetric measurements with manual mixing (panel B).

Stopped-flow kinetic measurements

Kinetics of PBI-S aggregation were measured by stopped-flow fluorescence experiments by mixing PBI-S solutions in 10 mM phosphate buffer pH 7.2 with NaCl solutions at increasing concentrations in the same buffer. The stopped-flow instrument was a SX-18 by Applied Photophysics apparatus. Acquisitions were performed by setting the detection for a time interval of 5 seconds and intensities acquired each 0.005 seconds. The excitation wavelength was set at 530 nm. Whole fluorescence signal was acquired after a passband filter at 545 nm.

The analysis of the kinetic traces was performed using MATLAB. The offset, amplitude, and exponential rate parameters were obtained by fitting the experimental data to the following exponential function:

$$y = y_0 + A(1 - e^{-k_{obs}t})$$

Spectrofluorometric kinetic measurements

In order to follow longer time courses (> 10 minutes), a spectrofluorometric measurement with manual mixing was used by using an Applied Photophysics RX2000 rapid kinetics spectrometer accessory on a fluorescence cuvette placed in a temperature-controlled apparatus (Jasco EHC-113) of a Jasco FP-8550 spectrofluorometer (Jasco Europe srl, Italy). The same solutions used in the stopped-flow measurements were used. Temperature was set at T = 25°C. The excitation and emission wavelengths were: λ_{exc} = 530 nm and λ_{em} = 560 nm, with bandwidths in excitation and emission set at 5 nm.

Stimulated Raman spectroscopy

In the experiments reported in the manuscript, both the Raman pump and the probe pulse were generated from a same laser source. Raman spectra acquired with an off-resonant Raman pump wavelength of 580 nm were obtained using a Ti:sapphire laser emitting 50 fs pulses centered at 800 nm at 1 kHz repetition rate. The PP was synthesized by focusing a small fraction (~1 μJ) of the fundamental beam onto a 3 mm sapphire crystal, generating a broadband white-light continuum that enabled the detection of vibrational modes across the entire Raman response. For collecting resonant SRS spectra (RP wavelength of 448 nm), we employed a Yb:KGW source delivering 190 fs pulses centered at 1030 nm, with a 10 kHz repetition rate. In this case, the PP was synthesized by focusing the second harmonic of the fundamental onto a 6 mm sapphire crystal, generating a broadband white-light continuum over the UV-visible spectral region. In both cases, the RP was synthesized by non-linear spectral compression producing few-picosecond pulses with a narrow bandwidth of ~7 cm^{-1} FWHM at 580 nm (~10 cm^{-1} at 448 nm) and approximately 1 μJ energy per pulse.

The RP and PP were focused onto the sample in a noncollinear geometry (~5°). The transmitted probe pulse was spectrally dispersed by a monochromator and detected onto a charge-coupled

device (CCD) operating in single-shot acquisition mode at the same repetition rate of the laser. To isolate the SRS response, consecutive probe spectra were acquired by chopping the RP at half of the laser repetition rate (500 Hz for the Ti:Sa and 5 kHz for the Yb source).

Steady state kinetic analysis

Time courses obtained from stopped-flow measurements were fitted to single-exponential curves using the Applied Photophysics kinetic analyser software. Observed rates were thus plotted as a function of PBI-S concentration at each given NaCl concentration. The initial rates thus obtained (v_{obs} vs [PBI-S]) were plotted and fitted to a simple Michelis and Menten scheme:

$$v_{obs} = V_{MAX} \frac{[\text{PBI-S}]}{K_M + [\text{PBI-S}]}$$

from which V_{MAX} and K_M are obtained at each given NaCl concentration. A MATLAB least squares fitting routine was used.

Observed amplitudes were also plotted against [PBI-S] concentrations at each given NaCl concentration. Data points immediately indicate that, at stationary state, the observed curves are highly suggestive of an anti-cooperative polymerization process. The K2–K model describes the self-assembly process of supramolecular polymers through two fundamental stages: nucleation and elongation. This minimal thermodynamic framework has been widely employed to analyse systems where polymerization is triggered by the formation of a critical nucleus, followed by the stepwise addition of monomeric units as follows: aggregation proceeds via the reversible formation of a dimeric nucleus (characterized by the dimerization constant K2), followed by elongation (described by the elongation constant K). In this framework, the total concentration c is partitioned into monomer concentration c_M and aggregated species. The degree of aggregation f_p (or fraction of polymeric species), representing the fraction of monomers incorporated into aggregates, is expressed as a function of normalized concentration $K\cdot c_M$ and the cooperativity factor σ = K2 / K, where:

- c is the total concentration of the compound (all the monomers, free and in polymers)
- c_M is the free monomer concentration
- c - c_M is the amount of monomeric units sequestered in aggregates (comprising dimers)
- $x = K\cdot c_M$ is a dimensionless variable.

Then, $f_p(x)$ as a function of x is obtained:

$$f_p = \frac{c - c_M}{c} = \frac{\sigma c_M \cdot \frac{2x - x^2}{(1-x)^2}}{c}$$

By substituting x for c, the final analytical expression is obtained (Eq. 2 in the manuscript):

$$f_{\mathrm{p}}(\mathrm{x}) = \frac{\sigma \cdot \dfrac{2\mathrm{x}-\mathrm{x}^2}{(1-\mathrm{x})^2}}{1-\sigma \cdot \dfrac{2\mathrm{x}-\mathrm{x}^2}{(1-\mathrm{x})^2}}$$

In the fitting procedure, the overall amplitude of the sigmoidal curves was allowed to float together with the σ values for each given NaCl concentrations, and, for clarity, the function f_p was plotted as a function of log(c). The elongation equilibrium constant K = K2 / σ was then obtained from the fitting.

Complete time dependent kinetic analysis

The experimental curves and their ODE-based fits (Figure 3) support the use of a truncated K2–K model that reduces to a non-linear reversible two step reaction:

$$\mathrm{M} + \mathrm{M} \underset{k_n^-}{\overset{k_n^+}{\rightleftharpoons}} \mathrm{M}_2 \qquad \text{followed by} \qquad \mathrm{M}_2 + \mathrm{M} \underset{k_e^-}{\overset{k_e^+}{\rightleftharpoons}} \mathrm{M}_3$$

Then, the system of ODEs becomes:

Monomer M_1 $\qquad \frac{dM_1}{dt} = -2k_n^+ M_1^2 + 2k_n^- M_2 - k_e^+ M_1 M_2 + k_e^- M_3$

Dimer M_2 $\qquad \frac{dM_2}{dt} = k_n^+ M_1^2 - k_n^- M_2 - k_e^+ M_1 M_2 + k_e^- M_3$

Trimer M_3 $\qquad \frac{dM_3}{dt} = k_e^+ M_1 M_2 - k_e^- M_3$

According to the law of mass conservation $c = c_M + 2c_{M2} + 3c_{M3}$, the observed fluorescence signal is considered proportional to the observed fluorescence amplitudes $A_1 c_M + A_2 c_{M2} + A_3 c_{M3}$. Initial guess for observed signal amplitudes was set considering $A_2 = 2A_1$ and $A_3 = 3A_1$. Numerical solutions of the ODE system required careful initialization due to stiffness introduced by the fast nucleation and slow elongation kinetics, for example by introducing small seed values for the dimer species (such as $c_{M2(0)}$ = 1 μM at 16 μM total concentration), realistically expected for the presence of a sizeable percentage of dimeric species in pure water within the concentration ranges used (2-32 μM). Under these premises, the curves were fitted by allowing floating the three amplitudes together with k_e^+ and k_e^- values.

Obs. Peak in PBI-S (shift cm^{-1})	Obs. Peak in PTC (shift cm^{-1})	Description	Mode assign. by Ong et al. (shift cm^{-1})	Mode assign. by Greiner et al. (shift cm^{-1})	Notes
1590–1580	1570	Perylene core C–C stretching along long axis	73-74<br>(1571.1-1590.6)	—	Strong perylene-like mode
1456		Naphthalene/perylene C–C–H in-plane bending	69<br>(1449.9)	$10a_g$<br>(1439)	Minor shift conserved across PBI-S (possible split with 1441 cm^{-1} in dimer state)
1380	1347	Symmetric C–H in-plane breathing or vinyl stretching (C–C stretching along long axis)	66<br>(1380.0)	—	Conserved mode across PBI-S
1300	1313<br>1287	Naphthalene stretching (C–H in-plane bending) or combination of C–H/C–C bending along long axis	59-60<br>(1279.3-1299.1)	$8a_g$<br>(1321)	Conserved mode across PBI-S and three-way split mode observed in PTC (possible conformational isomerism)
1090	1075	C–C bending and in-plane C–H deformations or vibrations of lateral groups	48<br>(1089.0)	—	Weak feature (possibly hindered by amine chains in PBI-S)
816	800	Low-frequency out-of-plane C–C–H bending or naphthalene deformation (C–C–C bending)	32-33-34<br>(796.9-819.1-819.8)	—	Average monomer feature and possible split at 800-735 cm^{-1}
545	550<br>524	Low-frequency out-of-plane biphenyl bending and in-plane C–C biphenyl stretching	16-19-20<br>(547.3-520.4-545.4)	$3a_g$<br>(560)	Weak aggregated feature and two-way split mode (possible molecule-molecule interactions)
411		Low-frequency in-plane skeletal torsions (C–C–C bending)	11<br>(416.8)	—	Weak monomer feature
	290	Low-frequency skeletal torsions (C–C–C bending)	8<br>(293.3)	—	Weak feature

Table S1. Main SRS peaks of PBI-S and PTC in comparison with theoretical and experimental perylene normal modes assignment
